# Immune response to *C. novyi*-NT immunotherapy

**DOI:** 10.1186/s13567-018-0531-0

**Published:** 2018-04-24

**Authors:** Amy E. DeClue, Sandra M. Axiak-Bechtel, Yan Zhang, Saurabh Saha, Linping Zhang, David Tung, Jeffrey N. Bryan

**Affiliations:** 1 Comparative Internal Medicine Laboratory, Department of Veterinary Medicine and Surgery, College of Veterinary Medicine, University of Missouri, 900 E. Campus Dr, Columbia, MO 65203 USA; 2 Comparative Oncology Radiobiology and Epigenetics Laboratory, Department of Veterinary Medicine and Surgery, College of Veterinary Medicine, University of Missouri, 900 E. Campus Dr, Columbia, MO 65203 USA; 3 Biomed Valley Discoveries, 4520 Main St, Kansas City, MO 64111 USA; 4 Present Address: College of Veterinary Medicine, University of Florida, 2015 SW 16th Avenue, Gainesville, FL 32608 USA

## Abstract

*Clostridium novyi*-NT (CVN-NT) spores germinate in hypoxic regions of tumors and have successfully cured induced neoplasia in mouse models and resulted in objective tumor responses in naturally developing neoplasia in the dog. The objective of this pilot, descriptive, prospective, clinical investigation, was to evaluate and describe the immune response to CNV-NT spores to better understand which immune pathways might play a role in the response to this bacteriolytic immunotherapy. Intratumoral injection of CNV-NT spores result in increased phagocytosis and NK cell-like function after treatment. Intravenous injection of CNV-NT spores resulted in increased LPS-induced TNF-α production, LTA-induced IL-10 production and NK cell-like function post-treatment. Increased NK cell-like function was sustained to 28 (intratumoral) or 56 (intravenous) days post-treatment, and increased phagocytic function was sustained to 28 days post-treatment suggesting that CNV-NT spores induce longer-term immune cell function changes. Future investigations evaluating long-term immune system changes and associations between immune function and tumor remission rates should include evaluation of these pathways.

## Introduction

For centuries it has been recognized that certain bacterial infections could induce tumor regression. In more modern times, the controlled use of bacteriolytic immunotherapy has been evaluated in both induced and spontaneous tumor models [1–3]. *Clostridium novyi*-NT (CNV-NT) spore immunotherapy has successfully cured induced neoplasia in mouse models and resulted in objective tumor responses in naturally developing neoplasia in the dog [1, 2, 4].

*Clostridium novyi* is an obligate anaerobic, spore forming bacterium found predominately in the soil that is associated with gas gangrene. CNV-NT typically causes pathological conditions through a combination of toxin release and the resulting inflammatory reaction. Alpha-toxin is one of the virulence factors produced by *C. novyi*. Alpha-toxin results in severe, painful edema which can progress to necrosis, refractory hypotension, leukocytosis, and visceral effusions. CNV-NT is derived from *C. novyi* wild type by heat treating the bacterial phage that carries the alpha toxin and serial passaging. This bacterium lacks the gene for the alpha-toxin [5]. Thus, toxicity is limited. However, CNV-NT maintains obligate anaerobic properties making it an ideal candidate bacteriolytic therapy for hypoxic solid tumors.

CNV-NT germinates in hypoxic regions of tumors yet does not germinate in other hypoxic regions including myocardial infarction. The exception to this is a report of a splenic abscess in a dog post-CNV-NT administration intravenously [2]. However, it is possible that this dog had an undetected metastatic lesion in the spleen that was the hypoxic focus of germination. After germination, CNV-NT secretes hydrolytic proteases and lipases which results in destruction of cancer cells and tumor regression in hypoxic regions. Oxygenated tissue regions near the outer rim of the tumor often remain unaltered by the local effects of CNV-NT, yet there is a 30% cure rate in CNV-NT spore treated mice [4]. In addition, after CNV-NT spore treatment, animals are protected against challenge with the same tumor cell line. Adoptive transfer of CD8^+^ cells from mice with neoplasia cured with CNV-NT into naïve mice prevents development of induced neoplasia [4]. This suggests that mechanisms other than isolated, direct effects of local CNV-NT infection contribute to positive outcomes, specifically, CNV-NT activates the innate immune system and primes specific anti-tumor CD8^+^ T cells. Since protection can be conferred from cured mice to naïve mice through adoptive transfer of CD8^+^ cells, it is likely that CNV-NT induces an immune response to the cancer cells in the inflammation following infection [4].

The objective of this pilot, descriptive, prospective, clinical investigation, was to evaluate and describe the immune response to CNV-NT spores to better understand which immune pathways might play a role in the response to this immunotherapy. To accomplish this goal, plasma immune biomarkers and immune cell function over time were measured in dogs with naturally developing, treatment-naïve neoplasia prior to administration of CNV-NT spores and then after either intravenous or intratumoral administration of CNV-NT spores.

## Materials and methods

### Population

Client owned dogs presented to the University of Missouri Veterinary Health Center with histologically or cytologically confirmed soft-tissue sarcoma, oral or cutaneous malignant melanoma, oral squamous cell carcinoma, or other cutaneous carcinomas were eligible for enrollment with informed client consent (IACUC protocol #7386). Eligibility criteria included tumors greater than 2 cm in diameter, tumor size and location where surgical excision with at least marginal resection was a viable option, and body weight of > 10 kg. Dogs were excluded if comorbidities were present that suggested survival expectation of less than 6 weeks, evidence of metastasis outside of the local draining lymph node, tumor location where abscess development would be catastrophic (e.g., CNS), persistent neutropenia or thrombocytopenia, grade 2 increases in plasma ALT, BUN or creatinine, administration of antimicrobial therapy within the 7 days preceding enrollment, concurrent infection requiring systemic antimicrobial therapy, chemotherapy, radiation therapy, or other immunotherapy within the 3 weeks preceding enrollment, pregnancy or potential pregnancy, enrollment in another clinical trial, cardiac disease severe enough that a balanced crystalloid solution administered at 4 mL/kg/h would likely induce congestive heart failure, and unavailability during the full study duration.

Baseline evaluations included medical history, physical examination, complete blood count, plasma biochemical profile, complete urinalysis, thoracic radiographs, and cytologic evaluation of the draining lymph bed if accessible. Prior to therapy, tumors were evaluated using PET/CT to evaluate tumor size, estimate glucose metabolism with [^18^F]FDG (data to be published in a separate manuscript), and estimate relative tumor hypoxia using [^64^Cu]ATSM (data to be published in a separate manuscript).

### Treatments

In the first cohort of dogs (July 2012–June 2013) the dogs received CNV-NT spores by intravenous (IV) administration on day 0. In the second cohort of dogs (July 2013–December 2013) the dogs received CNV-NT spores by intratumoral (IT) administration on day 0.

#### Intravenous administration

CNV-NT (1 × 10^9^) spores were administered in 50 mL of physiologic saline through an intravenous catheter. This dosage was determined based on a previous phase I clinical trial in dogs [1]. Initially, 5 mL was administered over 2 min; dogs’ vital parameters were monitored for 30 min. If the dog’s vital parameters were within 10% of baseline after 30 min, the remaining 45 mL was administered over a 5 min time period. Following completion of CNV-NT spore infusion, intravenous fluids were administered for 2 h and dogs were discharged to their owners if no signs of a reaction was noted.

#### Intratumoral administration

CNV-NT was provided in 3 mL of saline at a dose of 1 × 10^8^ spores based on previously described dosing in dogs [1]. Following sedation, the tumor was clipped and aseptically prepped, and CNV-NT spores were administered directly into the tumor with 0.75 mL injected into four total sites evenly distributed within the tumor by prior established spatial pattern. Vital parameters were monitored for 3 h following injection.

### Sample collection and processing

For the CNV-NT IV group, blood was collected at baseline (pre-treatment) and then at 7, 14 and 28 days. For the CNV-NT IT group, blood was collected at baseline (pre-treatment) and then 2, 30 and 56 days post-treatment. Blood was collected from the jugular vein and placed in either EDTA, sodium heparin, lithium heparin or evacuated tubes for immunologic evaluation. Lithium heparinized blood was immediately cooled and centrifuged within 1 h of collection. Plasma or serum was collected and stored at −80 °C for batch analyses of immune proteins. The remainder of the blood was processed immediately for PBMC isolation, phagocytic function and leukocyte cytokine production assays as indicated below.

### Plasma immune biomarkers

Lithium heparinized plasma was collected by centrifugation at 400 *g* for 15 min and then stored at −80 °C for batch analysis. Thirteen immune markers: IL-6, CXCL-8, IL-2, IL-7, TNF-α, GM-CSF, IL-18, CXCL-1, CXCL-10, IL-15, IL-10 and MCP-1 were evaluated in undiluted plasma as previously described using validated canine specific multiplex bead-based, ELISA assay (Millipore Sigma, Burlington, MA, USA), a MAGPIX Multiplexing instrument and analyzed using MILLPLEX Analyst 5.1 software [6]. Serum HMGB-1 (IBL International, Toronto, ON, Canada) and CRP (ICL Inc., Portland, OR, USA) were measured using commercially available ELISAs in accordance with the manufacturer’s recommendations as described previously [7, 8].

### Immune cell function

Immune cell function was assessed by evaluating granulocyte phagocytic and respiratory burst function, leukocyte cytokine production capacity and NK-like cell function.

Phagocytic function was determined using the Phagotest^®^ commercial test kit (Orpegen Pharma, Heidelberg, Germany) which evaluates phagocytosis of FITC-labeled, opsonized *Escherichia coli.* The assay has been previously validated in dogs and was performed as previously described [9, 10]. Samples were analyzed by flow cytometry using the CyAn ADP flow cytometer (Beckman Coulter, Brea, CA, USA) and associated data analysis software (Summit V 5.2.0.7477, Brea, CA, USA) within 30 min and a minimum of 15 000 events were recorded for each sample. DNA stain positive cells were gated and placed on a forward and side scatter plot. Phagocytes were identified using standard forward and side scatter characteristics. Then, FITC positive phagocytes were identified on a histogram. Both the relative number of *E. coli*-positive cells as well as the mean fluorescence intensity (MFI) of positive cells indicating the number of bacteria per cell were recorded.

The quantification of oxidative burst of PMNs was performed using a Phagoburst^®^ kit (ORPEGEN Pharma, Heidelberg, Germany) which evaluates *E. coli* and PMA—induced oxidative burst using dihydrorhodamine 123 as a fluorogenic substrate. The assay has been previously validated in dogs and was performed as previously described [9, 10]. Samples were analyzed by flow cytometry using the CyAn ADP flow cytometer and associated data analysis software within 30 min and a minimum of 15 000 events were recorded for each sample. DNA stain positive cells were gated and placed on a forward and side scatter plot. Phagocytes were identified using standard forward and side scatter characteristics. Then, a FLI histogram was used to identify positive phagocytes. The percentage of positive cells indicating recruitment and the MFI indicating the intensity of oxidative burst were recorded.

Leukocyte cytokine production capacity was determined by stimulating whole blood with lipopolysaccharide (LPS) from *E. coli* 0127:B8 (final concentration, 100 ng/mL; Sigma-Aldrich, St. Louis, MO, USA), lipoteichoic acid (LTA) from *Streptococcus faecalis* (final concentration, 1000 ng/mL; Sigma-Aldrich), or phosphate buffered saline (PBS; unstimulated control) and then measuring cytokine concentrations in the cell culture supernatant as previously described [11]. Blood was diluted 1:2 with media and samples were cultured on 12 well plates with LPS, LTA or PBS and then incubated for 24 h at 37 °C in 5% CO_2_. Cell supernatant was collected at end of incubation and stored in −80 °C for analysis. Quantification of TNF-α, IL-6 and IL-10 was accomplished using a canine specific multiplex bead-based, ELISA assay (Millipore Sigma) and a MAGPIX Multiplexing instrument as stated for the plasma immune markers.

NK-like cell function was determined using a thyroid adenocarcinoma cytotoxicity assay as previously described [12]. Canine thyroid adenocarcinoma (CTAC) cells were used as target cells. Prior to the assay, CTAC cells were labeled with 3 mM green fluorescent 3,3′-dioctadecyloxacarbocyanine (DiO) for 20 min at 37 °C in 5% CO_2_. Cytotoxicity of cancer cells was assessed by co-incubating PBMC with DiO-labeled CTAC cells for 24 h at 37 °C with 5% CO_2_. Cells were comingled in different PBMC to DiO-CTAC cell ratios: 1:1, 10:1, 25:1 and 50:1. Single cell population of PBMC or DiO-CTAC were used as controls. At end of incubation, cells were incubated with propidium iodide (PI). Samples were analyzed using the CyAn ADP flow cytometer and associated data analysis software. A minimum of 10 000 events were recorded for each sample. Data were analyzed as previously described [12]. Briefly, the CTAC cells were gated on a forward/side scatter plot and then applied to a plot comparing DiO and PI. DiO and PI positivity were determined using unstained cells as controls. Cells positive for DiO and PI were defined as dead CTAC cells. Baseline cell death was established using DiO/PI stained CTAC cells alone. The NK-like cell killing index was calculated by dividing the  % death from the PBMC + CTAC cell mixture by the CTAC cells alone.

### Statistical analysis

Statistical analysis was accomplished using SigmaPlot software (SigmaStat; Systat Software Inc., San Jose, CA, USA). Descriptive statistics were used to illustrate changes in immune function over time. Within each treatment group, observations at the three post-treatment time points were compared to baseline using a repeated measures ANOVA with Fisher least significant difference method for post hoc testing as appropriate. A Bonferroni correction was applied when appropriate to avoid multiple comparison testing limitations. A *P* value of < 0.05 was considered significant. Data are presented as mean and standard deviation unless otherwise noted.

## Results

Twenty dogs were enrolled, 10 dogs received IV and 10 dogs received IT administration of CNV-NT spores. The median age of dogs enrolled was 9.5 years (range 5–13 years, age of one dog unknown) and the median weight was 28.2 kg (range 14.4–70 kg). The sexes included intact male (*n* = 2), neutered male (*n* = 10), and spayed female (*n* = 8). Breeds of dogs enrolled were Labrador retriever (*n* = 5), Golden retriever (*n* = 3), Husky (*n* = 3), and one each of the following breeds: Standard Poodle, American Staffordshire terrier, English setter, Pug, Beagle, Bloodhound, Border Collie, Pointer, and mixed breed. Tumor types were soft tissue sarcoma (*n* = 10), oral sarcoma (*n* = 6), oral melanoma (*n* = 3), and apocrine gland adenocarcinoma (*n* = 1). One dog in the IV group was excluded from analysis due to development of gastric dilation and volvulus 9 days post-infusion, necessitating surgery and subsequent antibiotic therapy. This dog was withdrawn from trial and 19 dogs were included in the immunologic analysis.

**Figure 1:**
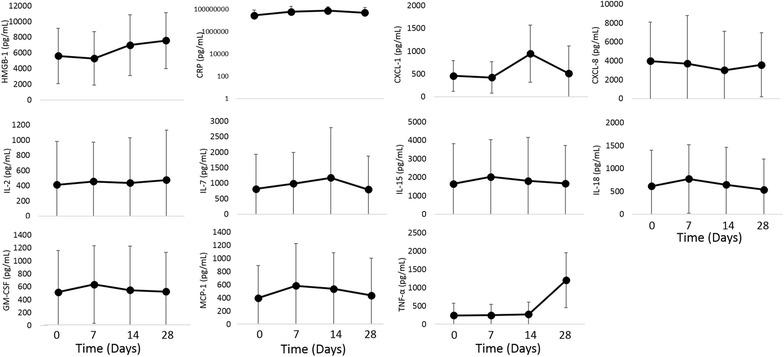
**Serum/plasma immune biomarkers after IV administration of CNV-NT spores.** Mean ± SD of serum HMGB-1 and CRP and plasma CXCL-1, CXCL-8, IL-2, IL-7, IL-15, IL-18, GM-CSF, MCP-1 and TNF-α at time 0 (pretreatment), and then at 7, 14 and 28 days post IV CNV-NT spore administration.

**Figure 2:**
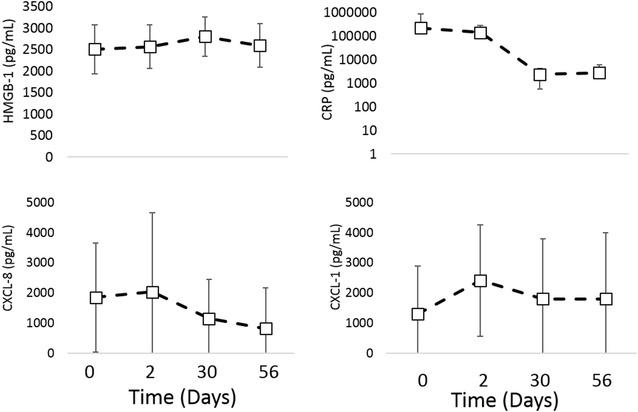
**Serum/plasma immune biomarkers after IT administration of CNV-NT spores.** Mean ± SD of serum HMGB-1 and CRP and plasma CXCL-1 and CXCL-8 at time 0 (pretreatment), and then at 2, 30 and 56 days post IT CNV-NT spore administration.

Serum HMGB-1 and CRP and plasma IL-6, CXCL-8, IL-2, IL-7, TNF-α, GM-CSF, IL-18, CXCL-1, CXCL-10, IL-15, IL-10, and MCP-1 were measured as markers of systemic immune system activation. Plasma IL-6, IL-2, IL-7, IFN-γ, TNF-α, GM-CSF, IL-18, CXCL-10, IL-15, IL-10 and MCP-1 concentrations fell below the assay’s limit of detection for greater than 50% of the samples for dogs treated with IT CNV-NT spores, and thus these markers were not analyzed (data not shown). Plasma IL-6, IL-10 and CXCL-10 concentrations fell below the limit of detection of the assay for greater than 50% of the samples for dogs treated with IV CNV-NT spores and thus these markers were not analyzed (data not shown). After Bonferroni correction, there were no significant differences in serum HMGB-1 and CRP and plasma CXCL-8, IL-2, IL-7, TNF-α, GM-CSF, IL-18, CXCL-1, IL-15 and MCP-1 before and after treatment in dogs that received IV CNV-NT spores (Figure 1). There was no difference in serum HMGB-1, CRP and plasma CXCL-1 or CXCL-8 in dogs that received IT CNV-NT spores (Figure 2).

**Figure 3:**
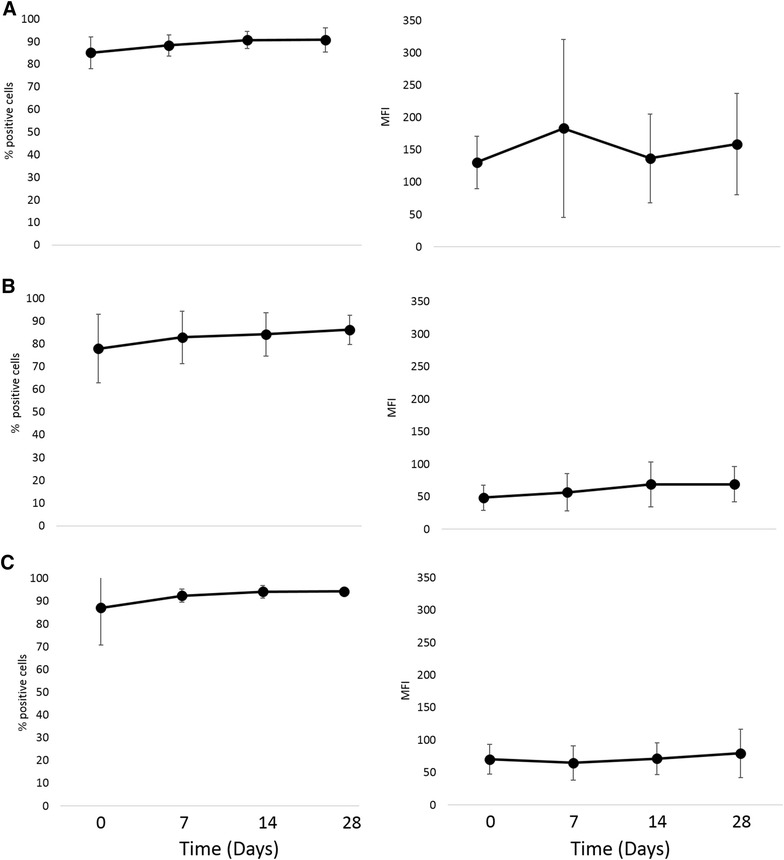
**Phagocytic function and respiratory burst function after IV administration of CNV-NT.** Mean ± SD of the **A** percent cells undergoing phagocytosis of opsonized *E. coli* (% positive cells) and the quantity of bacteria phagocytized (MFI), **B** the percentage of cells undergoing respiratory burst in response to *E. coli* (% positive cells) and the intensity of the respiratory burst (MFI), **C** the percentage of cells undergoing respiratory burst in response to PMA (% positive cells) and the intensity of respiratory burst (MFI) at time 0 (pretreatment), and then at 7, 14 and 28 days post IV CNV-NT spore administration.

**Figure 4:**
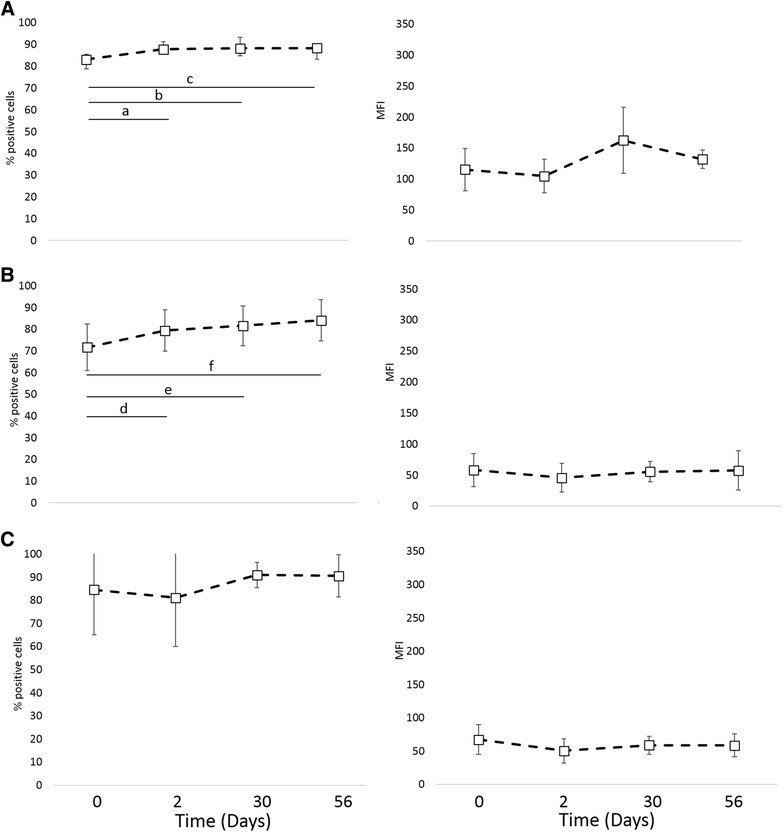
**Phagocytic function and respiratory burst function after IT administration of CNV-NT.** Mean ± SD of the **A** percent cells undergoing phagocytosis of opsonized *E. coli* (% positive cells) and the quantity of bacteria phagocytized (MFI), **B** the percentage of cells undergoing respiratory burst in response to *E. coli* (% positive cells) and the intensity of the respiratory burst (MFI), **C** the percentage of cells undergoing respiratory burst in response to PMA (% positive cells) and the intensity of respiratory burst (MFI) at time 0 (pretreatment), and then at 2, 30 and 56 days post IT CNV-NT spore administration. There was a significant difference in the percent cells undergoing phagocytosis between baseline and 2 days (*P* = 0.009) (a), 30 days (*P* = 0.006) (b) and 56 days (*P* = 0.006) (c) and the percent cells undergoing *E. coli*-induced respiratory burst at 2 days (*P* = 0.008) (d), 30 days (*P* < 0.001) (e) and 56 days (*P* < 0.001) (f).

**Figure 5:**
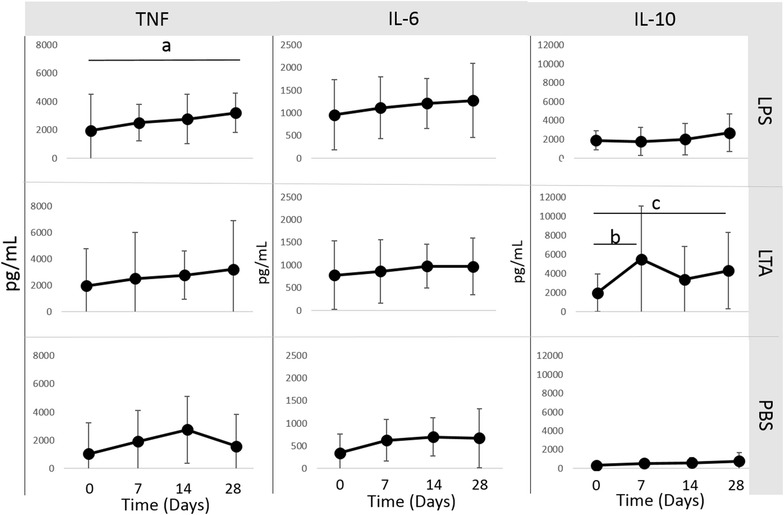
**Leukocyte cytokine production after IV administration of CNV-NT.** Mean ± SD of TNF-α, IL-6 and IL-10 production from canine blood after lipopolysaccharide (LPS), lipoteichoic acid (LTA) or phosphate buffered saline (PBS, control) stimulation at time 0 (pretreatment), and then at 7, 14 and 28 days post IV CNV-NT spore administration. There was significantly greater LPS-induced TNF-α production at 28 days (*P* = 0.002) (a) and LTA-induced IL-10 production at 7 days (*P* = 0.009) (b) and 28 days (*P* = 0.041) (c) compared to baseline.

**Figure 6:**
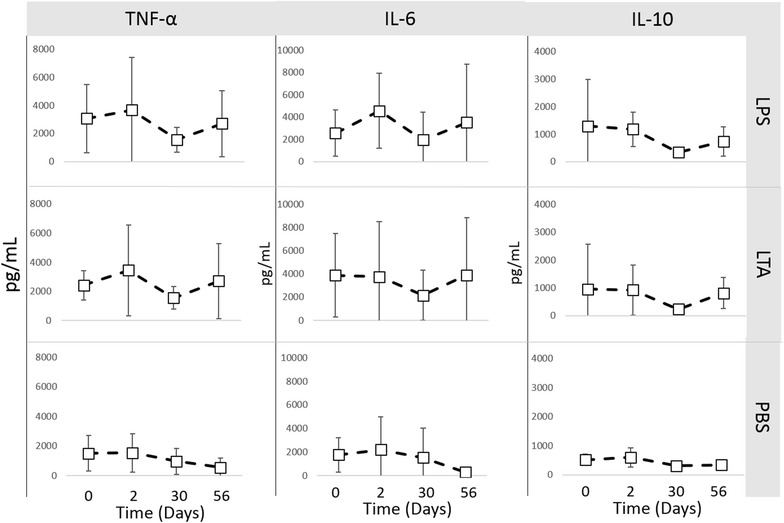
**Leukocyte cytokine production after IT administration of CNV-NT.** Mean ± SD of TNF-α, IL-6 and IL-10 production from canine blood after lipopolysaccharide (LPS), lipoteichoic acid (LTA) or phosphate buffered saline (PBS, control) stimulation at time 0 (pretreatment), and then at 2, 30 and 56 days post IT CNV-NT spore administration.

**Figure 7:**
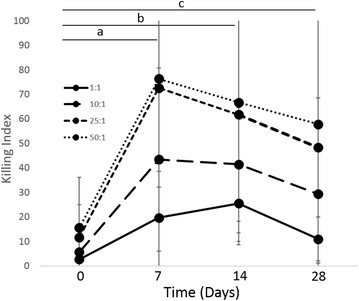
**NK cell-like function of PBMCs after IV administration of CNV-NT.** Mean ± SD of NK cell-like function of PBMCs at time 0 (pretreatment), and then 7, 14, and 28 days post IV CNV-NT spore administration when evaluated in ratios of PBMC:CTAC cells of 1:1, 10:1, 25:1 and 50:1. There was a significant difference between day 7 (*P* < 0.012 for all ratios) (a), day 14 (*P* < 0.005 for all ratios) (b) and day 28 (*P* < 0.048 for all ratios) (c) and baseline.

**Figure 8:**
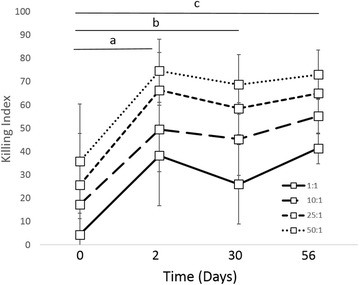
**NK cell-like function of PBMCs after IT administration of CNV-NT.** Mean ± SD of NK cell-like function of PBMCs at time 0 (pretreatment), and then at 2, 30 and 56 days post IT CNV-NT spore administration when evaluated in ratios of PBMC:CTAC cells of 1:1, 10:1, 25:1 and 50:1, respectively. There was a significant difference between day 2 (*P* < 0.001 for all ratios) (a), day 30 (*P* < 0.01 for all ratios) and day 56 (*P* < 0.002 for all ratios) (c) and baseline.

Phagocytic function, respiratory burst function, stimulated cytokine production and NK-cell like function were evaluated to assess immune cell function. There was no difference in phagocytosis or respiratory burst function after treatment with IV CNV-NT spores (Figure 3). In dogs treated with IT CNV-NT spores, the percent phagocytosis of opsonized *E. coli* was significantly greater at 2 days (*P* = 0.009), 30 days (*P* = 0.006) and 56 days (*P* = 0.006) compared to baseline (Figure 4A). Additionally, dogs treated with IT CNV-NT spores had significantly greater percentage of cells with *E. coli*-induced respiratory burst at 2 days (*P* = 0.008), 30 days (*P* < 0.001) and 56 days (*P* < 0.001) compared to baseline (Figure 4B). LPS-induced TNF-α production was significantly greater at 28 days (*P* = 0.002) than baseline in the dogs that received IV CNV-NT spores (Figure 5). Additionally, dog that received IV CNV-NT spores had significantly greater LTA-induced IL-10 production at 7 (*P* = 0.009) and 14 days (*P* = 0.041) compared to pre-treatment (Figure 5). There were no differences in leukocyte cytokine production capacity after IT CNV-NT spores administration (Figure 6). NK cell-like function significantly increased from baseline at day 7 (*P* < 0.012 for all ratios), day 14 (*P* < 0.005 for all ratios) and day 28 (*P* < 0.048 for all ratios) (Figure 7) after IV CNV-NT spore administration. Likewise, after IT CNV-NT spore administration, NK cell-like function was significantly increased from baseline at day 2 (*P* < 0.001 for all ratios), day 30 (*P* < 0.01 for all ratios) and day 56 (*P* < 0.002 for all ratios) (Figure 8).

## Discussion

In this study, we evaluated the immune response in dogs with naturally developing neoplasia that received either intratumorally or intravenously administered CNV-NT spores. The goal of this study was not to compare immune changes between IV and IT CNV-NT spore treatment or directly correlate immune response to tumor response. Rather, we were interested in how each of these administration methods altered the immune system independently. For this reason, we selected sample collection days based on the expected germination time and the expected time for the onset of inflammation. The key objective was to study the relatively longer-term immune response, and not just acute inflammatory changes or specific anti-tumor responses, thus samples were not collect to confirm germination to avoid altering the tumor microenvironment. We found that intratumoral injection of CNV-NT spores resulted in increased phagocytosis and NK cell-like function after treatment. When CNV-NT spores were administered intravenously, there was an increase in LPS-induced TNF-α production, LTA-induced IL-10 production and NK cell-like function post-treatment.

There is a paucity of information related to the immunologic response to CNV-NT germination in tumors in animals with neoplasia. Previous investigators have used the local inflammatory response and the development of fever as markers of immune response [2, 4]. Other investigators have noted that after CNV-NT germinates in canine tumors and rat glioblastoma, there is development of abscessation with neutrophilic inflammation [9, 13]. Further, administration of CNV-NT spores intravenously result in fever within 24 h of administration in the dog [9]. Yet, pre-treatment with diphenhydramine prevents the development of fever suggesting the fever is a manifestation of a hypersensitivity reaction, not a manifestation of infection [9]. However, fever was noted in some dogs at the time of abscess development. While these markers suggest the immune system plays a role in the host response to CNV-NT, they do not give insight into any persistent immune changes. Given that mice with tumors previously cured by CNV-NT were protected against challenge with the same tumor, it is likely there are persistent immune changes induced by CNV-NT [4].

Phagocytic function, respiratory burst function, and stimulated cytokine production capacity are often deficient in dogs with cancer [9, 10, 12, 14]. Here, we demonstrated that administration of CNV-NT spores IT increased phagocytic function and administration of CNV-NT spores IV increased cytokine production capacity. These alterations might serve to potentiate immune function and promote endogenous tumor cell recognition and clearance, and thus could be one mechanism of CNV-NT spore immunotherapy. In future larger scale clinical trials, it would be interesting to evaluate the association between increased phagocytic function, cytokine production capacity and tumor control.

NK cells are important for immune system surveillance for neoplastic or dysplastic cells. They have direct cytotoxic effects on neoplastic cells. In this study, both intravenous and intratumoral administration of CNV-NT spores resulted in a sustained increase in NK cell—like function. IL-2, IL-12, IL-15, IL-18 and IL-21 are known to be involved with NK cell development, activation and survival. Unfortunately, reliable, validated assays for canine specific IL-12 and IL-21 were not available at the time this study, so they were not evaluated. The plasma concentrations of IL-2, IL-15, and IL-18 did not show any changes after treatment with CNV-NT. The potential explanations for this includes the timing of sample collection not coinciding with peak cytokine production or that cytokine might have been produced locally with minimal systemic penetration leading to plasma concentrations falling below the assays’ limits of detection. Additionally, it is possible that other, unmeasured cytokines or inflammatory signals are involved in NK cell activation in the dog. Thus, it is unclear if cytokine signaling is involved with the observed increase in NK cell-like function. It would be interesting to see if there is an association between increased NK-cell like function and tumor control in future studies as well as assessing local inflammatory mediator production.

Dogs develop abcessation approximately 5 days after intravenous spore administration [2]. It was previously reported that following IV CNV-NT spore administration, the blood of all animals were positive for CNV-NT culture for 3 h, then the rate of detection dropped off until reaching 2/6 positives at 14 days [2]. Thus we elected to look at the immune response approximately 2 days post-expected germination, and then at 2 and 4 weeks to see if the immune changes were sustained. For the IT group, it was hypothesized that germination would be rapid, within 6 h, so initial samples were collected at 2 days post-administration. Then we looked at 4 weeks and 56 days for a longer-term assessment of immune parameter changes.

This pilot study for examining the dynamic immune response in dogs with naturally developing neoplasia had several limitations. First, the canine population studied was heterogeneous and the dogs had a variety of tumor types which resulted in increased variability among dogs and reduced our ability to identify immunologic changes over time. Dogs were not selected based on pre-treatment immune system function so it is possible that variations in immunocompetence altered the immediate and long-term immune response to CNV-NT spores. With the two different delivery methods and different sampling schedules, a direct comparison between the two treatment delivery methods was not possible. It would be interesting to compare the two delivery methods in the future. We also did not correlate the immune response with the degree of tumor infection. This measure was purposefully left out since enumerating the bacteria would have required removal of the tumor resulting in alteration of the long-term immune response. Nevertheless, it should be noted that we did not document a direct association between bacterial germination and the immune response described here and it is possible the documented immune changes are in response to CNV-NT spores, not true infection. Finally, immune response to CNV-NT was not correlated with tumor response so it is not known if the observed immunologic changes would result in tumor control.

Injection of CNV-NT spores induce an immune response in dogs with naturally developing neoplasia. Specifically, intratumoral injection of CNV-NT spores result in increased phagocytosis and NK cell-like function after treatment. When CNV-NT spores were administered intravenously, there was an increase in LPS-induced TNF-α production, LTA-induced IL-10 production and NK cell-like function post-treatment. Of these, increased NK cell-like function was sustained to 28 (IT) or 56 (IV) days post-treatment, and increased phagocytic function was sustained to 28 days suggesting that administration of CNV-NT spores induces longer-term immune cell function changes. Future investigations evaluating long-term immune system changes and associations between immune function and tumor remission rates should include evaluation of these pathways.

## Abbreviations

CTAC: canine thyroid adenocarcinoma
CVN-NT: *Clostridium novyi*-NT
DiO: dioctadecyloxacarbocyanine
IT: intratumoral
IV: intravenous
LPS: lipopolysaccharide
LTA: lipoteichoic acid
MFI: mean fluorescence intensity
PBS: phosphate buffered saline
PI: propridium iodide
